# Early involvement in friendships predicts later plasma concentrations of oxytocin and vasopressin in juvenile rhesus macaques (*Macaca mulatta*)

**DOI:** 10.3389/fnbeh.2014.00295

**Published:** 2014-08-28

**Authors:** Tamara A. R. Weinstein, Karen L. Bales, Nicole Maninger, Caroline M. Hostetler, John P. Capitanio

**Affiliations:** ^1^California National Primate Research Center, University of CaliforniaDavis, CA, USA; ^2^Department of Behavioral Neuroscience, Oregon Health and Science UniversityPortland, OR, USA

**Keywords:** affiliation, friendship, oxytocin, rhesus macaque, social behavior, vasopressin

## Abstract

The neuropeptides oxytocin (OT) and arginine vasopressin (AVP) are involved in social bonding in attachment relationships, but their role in friendship is poorly understood. We investigated whether rhesus macaques’ (*Macaca mulatta*) friendships at age one predicted plasma OT and AVP at two later time points. Subjects were 54 rhesus macaques at the California National Primate Research Center (CNPRC). Blood was drawn during a brief capture-and-release in the home cage, and plasma assayed for OT and AVP using an enzyme immunoassay (EIA). Separate linear mixed models for each sex tested the effects of dominance rank, age, sampling time point, housing condition, parturition status, two blood draw timing measures, and five friendship types: proximity friendships, play friendships, reciprocal friendships (a preference for a peer that also preferred the subject), multiplex friendships (friendships displayed in more than one behavioral domain), and total number of friendships. Females’ number of reciprocal and play friendships at age one significantly predicted later OT; additionally, these two friendship types interacted with rank, such that high-ranking females with the fewest friendships had the highest OT concentrations. Friendship did not predict later OT levels in males, however proximity, play, reciprocal, and total number of friendships predicted males’ plasma AVP. Play and total number of friendships also tended to predict AVP in females. Our results show that peripheral measures of neuroendocrine functioning in juvenile rhesus monkeys are influenced by early involvement in friendships. Friendships have an especially strong impact on an individual’s psychosocial development, and our data suggest OT and AVP as potential underlying mechanisms. Moreover, sex differences in the functioning of the OT and AVP systems, and their relation to friendship, may have important clinical implications for the use of OT as a therapeutic, as well as informing the social context in which it is administered.

## Introduction

Friendships are critically important for healthy development throughout the lifespan, and a lack of friends increases vulnerability to mental disorders such as depression, physical illnesses such as cardiovascular disease, and mortality (Cohen and Wills, 1985; House et al., 1988; Uchino et al., 1996; Thorsteinsson and James, 1999). Friendships are particularly vital for successful psychosocial adjustment in children, and greater involvement in friendships is associated with more positive school perceptions and performance gains in kindergartners (Ladd, 1990), decreased feelings of loneliness and depression in 3rd-6th graders (Nangle et al., 2003), lower levels of teacher reported maladjustment during adolescence (Waldrip et al., 2008), and decreased loneliness and self-reported victimization in adolescents suffering from social anxiety (Erath et al., 2010). Preadolescence may be an especially critical time period for the successful development of adult social skills and ties (Fullerton and Ursano, 1994), and the consequences of friendship formation during this period may be far reaching; for example, friended preadolescents report higher levels of self-worth in adulthood when measured 12 years later, while the absence of friendship is associated with psychopathological symptoms in adulthood (Bagwell et al., 1998). It is notable that while having high quality, supportive relationships both with attachment figures (i.e., parents) and with peers impacts children’s psychosocial development, having high quality peer friendships can buffer the negative consequences experienced by children with low parental support (Rubin et al., 2004).

Nonhuman primate friendships are characterized by many of the same features as human friendships (Smuts, 1985; Silk, 2002), and are linked to many similarly beneficial consequences. Having strong, long-lasting friendships enhances longevity, infant offspring survival, and offspring longevity (Silk et al., 2003, 2009, 2010), and friends buffer individuals’ behavioral and physiological responses to stressors (Higley et al., 1992; Gust et al., 1994; Boccia et al., 1997; Beehner et al., 2005; Crockford et al., 2008; Wittig et al., 2008). As with human children, befriending peers is vital for nonhuman primate youngsters to establish independence from their mothers and successfully integrate themselves into the wider network of their social group (Hinde and Spencer-Booth, 1967), as well as to learn social skills necessary for successfully navigating the complex relationships that they will encounter as adults (Poirier and Smith, 1974; Joffe, 1997). In rhesus macaques (*Macaca mulatta*), friendships are clearly identifiable at least as early as 1 year of age, and, like children’s friendships, their formation and persistence are influenced by individual-level characteristics such as personality and relationship-level qualities such as reciprocity (Weinstein and Capitanio, 2008, 2012). Juvenile rhesus monkeys reap short-term benefits from their friends, such as an attenuated distress response in a novel context (Higley et al., 1992), as well as long-term advantages such as facilitating dominance rank acquisition during adolescence (de Waal and Luttrell, 1985; Datta, 1988). Rhesus macaques have long been regarded as a valuable model system in which to examine socioemotional development, due to the cognitive abilities, physiological and neuroanatomical characteristics, and socioemotional complexity which they share with humans (Suomi, 2005; Capitanio and Emborg, 2008; Weinstein et al., 2008; Nelson and Winslow, 2009; Weiss et al., 2011), and may therefore be uniquely capable of filling the large gap that exists between human and rodent research on the neurobiological basis of affiliative relationships (Weinstein and Capitanio, 2005). The few studies thus far that have attempted to close this gap suggest that the neuropeptides oxytocin (OT) and arginine vasopressin (AVP) play a key role.

OT and AVP are nine-amino acid peptides produced in the hypothalamus and released into circulation via the posterior pituitary (Zingg, 2002). OT and AVP differ only by two amino acids (Sofroniew, 1985) and can bind to each other’s receptors (Gimpl and Fahrenholz, 2001). In both nonhumans and humans, OT plays a role in mother-infant interactions (Pedersen and Prange, 1979; Feldman et al., 2010b), father-infant interactions (Feldman et al., 2010a), and adult romantic attachments (Carter et al., 1995; Cho et al., 1999; Gordon et al., 2008; Scheele et al., 2012), and AVP underlies male pair-bonding (Winslow et al., 1993; Cho et al., 1999; Lim and Young, 2004; Jarcho et al., 2011) and parenting behavior (Wang et al., 1998; Bales et al., 2004b). Though OT and AVP are rarely studied in the context of non-attachment relationships such as friendships, they are strong candidates for several reasons. First, OT has previously been implicated in behaviors known to be important to friendship including trust (Zak et al., 2005; Kéri and Kiss, 2011; Zhong et al., 2012), generosity (Zak et al., 2007; Baumgartner et al., 2008), social support (Heinrichs et al., 2003; Buchheim et al., 2009), and reciprocal or synchronous behaviors (Atzil et al., 2012). Notably, in wild chimpanzees, grooming with a “bond partner” (defined by mostly positive dyadic social behaviors, such as grooming, coalitionary support, etc.) resulted in higher urinary OT levels following grooming, regardless of whether the bond partner was related or not (i.e., a friend; Crockford et al., 2013). Second, AVP has a role in enhancing the recognition of emotional states (Guastella et al., 2010) and in sex-specific perceptions of the friendliness of faces (Thompson et al., 2006). Finally, OT and AVP can have far-reaching developmental effects on behavior and vice versa (Carter, 2003; Bales and Perkeybile, 2012; Veenema, 2012). Pharmacological exposure to OT or AVP either at birth (Bales and Carter, 2003a,b; Bales et al., 2004a, 2007a,b; Gregory et al., 2013), later in the perinatal period (Stribley and Carter, 1999), or in the peri-adolescent period (Bales et al., 2013) can have long-term, organizing effects on social behaviors, anxiety behaviors, and neural systems. Conversely, early experiences, such as the quality of parental care received, can shape both humans’ and animals’ neuropeptide response (Bartz et al., 2010; Bales and Perkeybile, 2012; Veenema, 2012; Feldman et al., 2013). Given the impact of early parental experience on an offspring’s OT and AVP systems, it is likely that early peer experiences, particularly friendship involvement, would similarly influence later OT and AVP responses.

In the current study, we examined how friendships in yearling rhesus macaque males and females predicted plasma OT and AVP concentrations approximately 1–3 years later. Though many animal studies of OT and AVP have measured central concentrations or receptor distributions, studies of humans rely almost exclusively on peripheral measures, whose meaning is still being unraveled, but which may reflect responses to a range of stimuli, including social stimuli (Kenkel et al., 2012). It is likely that peripheral levels do not fully capture central levels (Kagerbauer et al., 2013), although they may be correlated under certain conditions (Landgraf and Neumann, 2004). It is important to continue to examine the utility and predictive value of peripheral peptide measures, because until non-invasive technology advances significantly, central measures in living humans are difficult to obtain. Because previous research has shown numerous sex differences in baseline neuropeptide concentrations (Kramer et al., 2002; Miller et al., 2013), regulation by gonadal hormones (Witt et al., 1991; De Vries and Villalba, 1997; Cushing et al., 2003), and sensitivity to exogenous stimulation (Carter, 2003, 2007), we hypothesized that we would find sex-specific associations between plasma OT, AVP, and friendship. As high peripheral levels of OT and AVP have been associated with improved social functioning in the case of children with autism spectrum disorders (Modahl et al., 1998; Green et al., 2001), we specifically predicted that having more friendships (and particularly higher-quality friendships) would be associated with high OT in females and high AVP in males. However, high plasma OT and AVP levels in adult humans may sometimes be associated with a social challenge, such as a distressed pair-bond relationship (Taylor et al., 2006, 2010); therefore an alternate prediction was that the stress associated with fewer social connections would result in higher levels of peptides later (presumably, higher OT in females and higher AVP in males). The most recent data suggest that OT and AVP levels may often exhibit complex, nonlinear relationships with social variables; a recent study with a very large sample showed a U-shaped relationship between OT levels and trust (Zhong et al., 2012). We therefore also tested whether friendship involvement in our subjects would demonstrate a non-linear relationship with neuropeptide levels measured later in development.

## Materials and methods

### Subjects and housing

Subjects were 54 rhesus macaques (29 males) that had participated in our previous study of yearling friendships (Weinstein and Capitanio, 2008; note that our original *N* = 57, however only 54 animals were available for blood sampling for the current study). Subjects were housed in half-acre (0.19 ha) outdoor breeding corrals at the California National Primate Research Center (CNPRC). Each corral housed approximately 90–150 animals of all age/sex classes year-round, and, like wild rhesus monkey troops, groups were organized around female-headed extended families (matrilines). Each corral contained several separate matrilines, which in turn typically comprised several generations of kin. Corrals were constructed of chain-link sides and top measuring 30.5 m wide × 61 m deep × 9 m high, a natural substrate floor, several wooden A-frame structures, PVC-coated perches, a variety of climbing devices, and several food hoppers. Primate laboratory chow was provided twice daily, fruit and vegetable supplements were provided twice weekly, and water was available ad libitum.

For the yearling behavioral data collection portion of the study, all subjects were housed in the outdoor breeding corrals described above. However, shortly before the first blood sample was collected, six subjects were removed from their natal groups due to social instability. Five of these animals were sampled while pair-housed in standard indoor living cages measuring 0.58 × 0.66 × 0.81 m, and one subject was sampled while living in an outdoor corncrib. Corncrib groups contained 1 adult male and 3–6 adult females and their offspring, and consisted of two outdoor cylindrical structures approximately 4 m in diameter constructed of chain-link fencing and connected by a rectangular passageway. Like the breeding corrals, corncribs contained PVC perches, climbing structures, and food hoppers. At the time the second blood sample was collected, 42 subjects were living in the outdoor breeding corrals, 8 had been relocated indoors, and 1 subject continued to live in a corncrib.

### Affiliative behavioral data collection

We previously described affiliative behavior data collection methods (Weinstein and Capitanio, 2008). Briefly, when subjects were approximately 1 year of age, we conducted 15 10-min continuous focal observations on each subject over a 10-week period during the mating season. Mating season in this population typically lasts from August through December. Subjects consisted of two birth cohorts, and were therefore observed in two separate years, in 2003 and 2004. Subjects’ mean age at the start of behavioral observations was 1.44 years. We observed subjects between 0800 and 1200 h, 4–5 days per week, and used a Psion Workabout handheld computer equipped with The Observer Mobile 3.0 (Noldus Information Technology, Wageningen, The Netherlands) to record frequencies and durations of proximity (within arm’s reach of another monkey), play (shoving, grabbing, slapping, chasing, pushing, wrestling and/or mouthing behavior accompanied by a play face (wide eyes, open mouth without bared teeth) or a loose, exaggerated posture and gait), nonaggressive physical contact, and grooming (picking through and examining the fur of another monkey, parting the fur with the hands or mouth). We recorded identities of all participants as well as the direction of initiation of all interactions. Inter-observer reliability exceeded 80% for all behaviors.

### Assessment of dominance ranks

Dominance relations among corral group members were regularly assessed by behavioral management staff at the CNPRC by observing the monkeys’ feeding order and direction of displacements and aggression both around and in the absence of temporarily concentrated food sources. Each corral contained a clear-cut linear dominance hierarchy between the different matrilines, such that all members of the highest-ranking matriline were dominant over all members of the second-ranking matriline, who in turn outranked all members of the third-ranking matriline, and so forth. We used the numerical dominance ranks derived by the staff for each adult female at the time of yearling behavioral data collection to classify matrilines as high-, middle-, or low-ranking (i.e., top, middle, or bottom one-third of the hierarchy). Since yearling rhesus macaques have not yet formalized their own ranks in the hierarchy, we classified them according to maternal rank.

### Blood sampling procedure

All available animals that had previously been subjects in the yearling affiliation study were sampled. We collected blood samples from subjects on two occasions: the first sample was obtained in October-November 2005 between 0115 h and 0230 h, and the second sample in July-August 2006 between 1000 h and 1100 h. All animal handling and blood sampling procedures were reviewed and approved by the Institutional Animal Care and Use Committee of the University of California, Davis. At the time the first sample was collected, 54 subjects were available for sampling: 48 subjects were still living in the outdoor breeding corrals, five were pair-housed in standard indoor living cages, and one was living in an outdoor corncrib. At the time the second blood sample was collected, 51 subjects were available: 42 subjects were housed in the outdoor breeding corrals, 8 had been relocated indoors, and 1 subject continued to live in a corncrib. Each outdoor-housed subject was net captured and manually restrained in their corral or corncrib by trained animal technicians, and a 1.0 ml sample of blood was drawn via femoral venipuncture. Subjects were released back into their corral or corncrib immediately following sample collection. A maximum of 3 animals per corral were sampled per day in a predetermined random order. Indoor-housed animals were restrained by use of an intra-cage squeeze mechanism. After collection, blood samples were transferred into sterile, vacuum-sealed tubes coated with heparin, which were placed on ice until being brought to the laboratory for storage and analysis. During blood sampling, an observer used a stopwatch to record the following time points: first entry into the outdoor cage or indoor room; if outdoors, the instant the animals was captured by the net; if indoors, the moment at which the technician approached the subject’s living cage; and the point at which the needle was withdrawn.

### Assays

After blood samples were brought to the laboratory, they were centrifuged at 4°C for 10 min to separate and extract plasma. Plasma was stored at −80°C until assay. We measured plasma concentrations of OT and AVP using a commercially prepared enzyme immunoassay (EIA) kit manufactured by Assay Designs (Ann Arbor, MI) and validated for use in rhesus macaques by Bales et al. (2005). Prior to assay, plasma was diluted with assay buffer using a 1:6 ratio for the OT assay, and a 1:8 ratio for the AVP assay. Intra-assay coefficients of variation were 4.24% (OT) and 5.16% (AVP), and the inter-assay coefficients of variation were 3.71% for OT and 2.13% for AVP. Assay sensitivity was 15.55 pg/ml for OT and 2.34 pg/ml for AVP; none of the samples fell below assay sensitivity.

### Statistical analysis

#### Quantifying friendships

We had previously calculated each subject’s friendships based on durations of affiliative interactions with same-age peers (see Weinstein and Capitanio, 2008). We divided each subject’s data into five 2-week periods (biweeks); during each biweek we had collected three 10-min focal samples per subject. We then calculated the total duration of affiliation initiated by each subject toward each peer during a given biweek as a percentage of total time observed. We entered each of these percentages into separate tables for each subject, with columns representing biweeks (*N* = 5), and rows representing all peer group-mates. Any peer(s) with whom the focal animal did not affiliate during a given biweek received a “0” cell value. For each biweek, we then calculated the percentage of time the subject would have spent affiliating with each peer if the interactions were distributed equally across all peers during that time period, by summing the actual values in each column (i.e., biweek) and dividing by the total number of rows (peers) to give the expected value of that subject’s affiliation with each peer. For example, if a subject with five peer group-mates spent 10% of her time affiliating with those peers during a given biweek, she would be expected to spend 2% of her time with each peer if she were interacting randomly. We then used a chi square test (*df* = 4) to compare the actual and expected percentages for each individual peer across the 5 biweeks. Subject X was considered to have initiated a friendship with animal Y for behavior Z when X’s actual scores were significantly greater than expected (*p* < 0.05). We calculated friendships for each affiliative behavior separately, and classified them according to behavioral content and quality, with reciprocal and multiplex friendships representing the highest quality friendships (Table 1). Because our subjects rarely, if ever, engaged in grooming and contact with peers, we did not consider grooming or contact friendships as standalone variables in our analyses, but we did factor them into the calculations of multiplex, reciprocal, and total number of friendships.

**Table 1:** **Yearling friendship classification**.

**Proximity Friendship**	A preference displayed by the subject toward a peer for proximity
**Play Friendship**	A preference displayed by the subject toward a peer for play
Multiplex Friendship*	A preference displayed by the subject for a peer in more than one behavioral domain (e.g., subject preferred the peer for both proximity and play)
Reciprocal Friendship*	A preference displayed by the subject for a peer that also preferred that subject, regardless of behavioral domain
**Total Number of Friends**	The total number of individually unique peers that were preferred by a subject, irrespective of behavioral domain

*Denotes higher quality friendships.

#### Relating friendships to neuropeptides

We used a repeated measures Linear Mixed Model analysis with an unstructured repeated covariance type in IBM SPSS Statistics version 21 to determine whether subjects’ friendships at age 1 (see Table 1) predicted plasma neuropeptide levels at two later points in development. We also included maternal dominance rank held at the time of behavioral data collection, sampling time point, birth cohort, parturition status, housing condition (living outdoors in a socially stable group, living outdoors in a socially unstable group, or living indoors), and blood sampling disturbance and draw times as independent variables. Blood sampling disturbance time was the number of seconds that elapsed between the time the door to the outdoor corral or indoor room was opened and the time the needle was withdrawn. Draw time was the number of seconds that elapsed between net capture and needle withdrawal for outdoor animals, and the number of seconds that elapsed between cage approach and needle withdrawal for indoor animals. Because OT and AVP’s relation to behavior has previously been found to differ according to sex in a number of studies (e.g., Bales et al., 2004c, 2007a, 2013), we computed the models separately for males and females. For each sex, we began with a model that included all independent variables, and used a backward elimination procedure to delete variables with a *p*-value ≥ 0.10 until all non-significant variables had been removed, aside from maternal dominance rank. Because our previous analyses revealed that subjects’ friendships varied significantly according to maternal dominance rank (Weinstein and Capitanio, 2008), we left this variable in as a control in our models. We then tested for interactions between maternal dominance rank and all significant friendship variables that remained in each model, and used backward elimination to delete non-significant interactions. Finally, we assessed whether friendship had a nonlinear relationship with neuropeptide levels by testing the significance of the quadratic function of the friendship variables that remained in each model. Table 2 lists the number of subjects in each categorical independent variable group.

**Table 2:** **Number of subjects in each categorical independent variable group: sample 1 (sample 2)**.

		Females	Males
**Total**		25 (25)	29 (26)
**Pregnant/Lactating**		10 (10)	N/A
	**High**	6 (6)	6 (6)
**Rank**	**Middle**	8 (8)	9 (9)
	**Low**	11 (11)	14 (11)
**Birth Cohort**	**2002**	14 (14)	14 (12)
	**2003**	11 (11)	15 (14)
**Housing Condition**	**Outdoor Stable**	17 (17)	17 (16)
	**Outdoor Unstable**	6 (7)	9 (3)
	**Indoors**	2 (1)	3 (7)

## Results

### Oxytocin model–females

**Figure 1:**
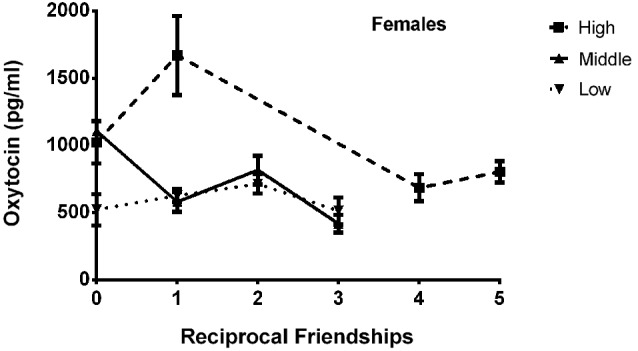
**There was a significant interaction between rank and reciprocal friendships (*F*_(2,14)_ = 10.083, *p* = 0.002), such that middle- and high-ranking females with the fewest reciprocal friendships had high OT levels**. Additionally, high-ranking females had the highest numbers of reciprocal friendships, and they also had high OT levels.

**Figure 2:**
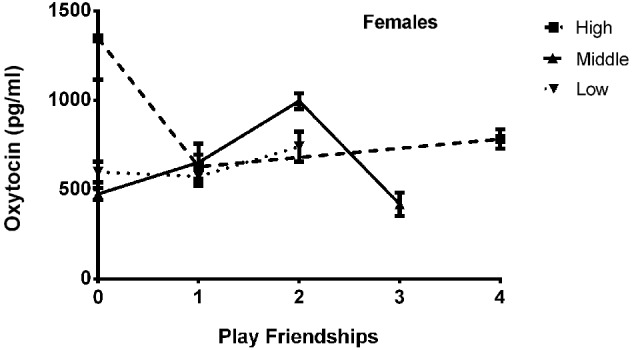
**There was a significant interaction between rank and the number of play friendships (*F*_(2,14)_ = 7.763, *p* = 0.005), such that high-ranking females with no play friendships had the highest OT levels, which decreased as the number of friendships increased**.

**Figure 3:**
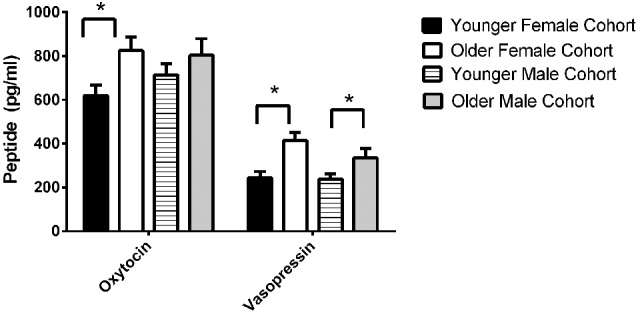
**We found significant birth cohort differences in OT for females, with older females having higher OT levels than younger females (*F*_(1,14)_ = 19.669, *p* = 0.001)**. We also found significant birth cohort differences in AVP for both females (*F*_(1,19)_ = 5.173, *p* = 0.035) and males (*F*_(1,19.245)_ = 5.275, *p* = 0.033). In both sexes, older animals had higher AVP concentrations than younger animals.

The final model for OT in females contained a number of significant friendship variables. The relationship between the number of reciprocal friendships and plasma OT was significantly described by a U-shaped quadratic function (*F*_(1,14)_ = 8.027, *p* = 0.013), such that overall, females with low or high numbers of reciprocal friendships had higher OT levels. Though rank by itself was not significant (*F*_(2,14)_ = 2.717, *p* = 0.101), there was a significant interaction between rank and reciprocal friendships (*F*_(2,14)_ = 10.083, *p* = 0.002; Figure 1), such that middle- and high-ranking females with the fewest reciprocal friendships had high OT levels. Additionally, high-ranking females who had the greatest numbers of reciprocal friendships also had high OT levels. The number of play friendships also significantly positively predicted plasma OT (*F*_(1,14)_ = 11.492, *p* = 0.004), and there was a significant interaction between rank and the number of play friendships (*F*_(2,14)_ = 7.763, *p* = 0.005; Figure 2). As shown in Figure 2, high-ranking females with no play friendships had the highest OT levels, which decreased as the number of friendships increased. Finally, cohort (the year that animals were born and therefore represents age) was significant, with older females having higher OT levels than younger females (*F*_(1,14)_ = 19.669, *p* = 0.001; Figure 3). We did not find sampling time point, parturition status, housing condition, or blood sampling disturbance or draw times to significantly predict later OT levels (all *p* > 0.1). Beta coefficients for independent variables reaching statistical significance are in Table 3.

**Table 3:** **Linear mixed model for females’ friendships and plasma oxytocin concentrations**.

Variable	*B*	Standard error
Birth cohort: 2002	348.37**	78.55
Birth cohort: 2003*^a^*	–	–
High Rank	128.81	155.83
Middle Rank*^a^*	–	–
Low Rank	−193.62	113.34
Play Friendships	224.76**	41.45
Reciprocal Friendships	−210.08*	80.77
Reciprocal Friendships^2^	−56.57*	19.97
High Rank × Play Friendships	−157.08*	61.79
Middle Rank × Play Friendships*^a^*	–	–
Low Rank × Play Friendships	−234.60**	61.95
High Rank × Reciprocal Friendships	314.44**	83.13
Middle Rank × Reciprocal Friendships*^a^*	–	–
Low Rank × Reciprocal Friendships	259.96**	67.05

^a^Reference category; *p < 0.05; **p < 0.01.

### Oxytocin model—males

Oxytocin was not related to friendship or any other independent variable for males.

### Vasopressin model—females

**Figure 4:**
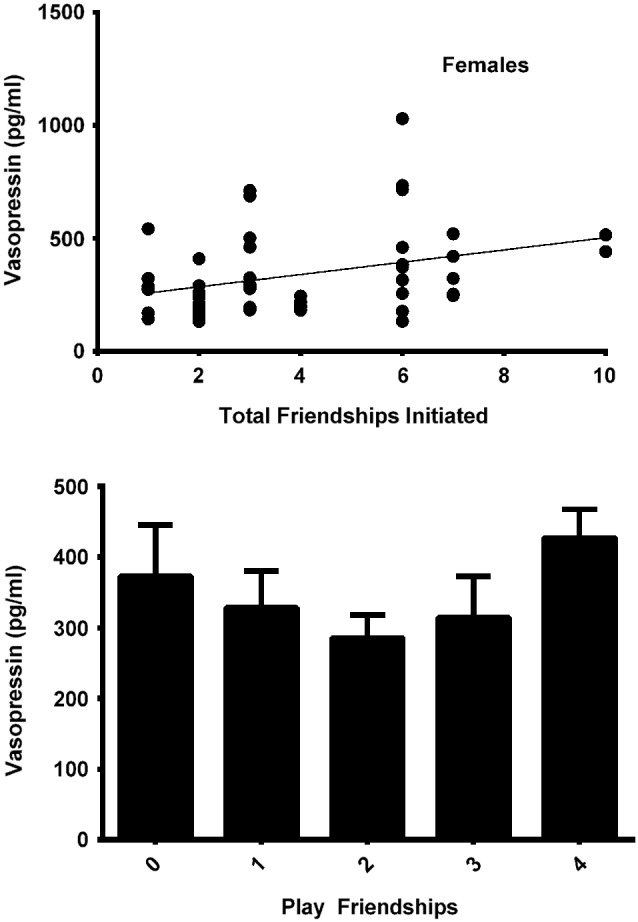
**Top panel: In females, the total number of friendships initiated tended to positively predicted AVP levels (*F*_(1,19)_ = 4.180, *p* = 0.055)**. Bottom panel: The total number of play friendships held by females tended to negatively predict AVP levels (*F*_(1,19)_ = 4.021, *p* = 0.059).

The final model for vasopressin in females contained two friendship variables that neared significance: a positive trend for the total number of friendships initiated by subjects (*F*_(1,19)_ = 4.180, *p* = 0.055; Figure 4, top panel), and a negative trend for the total number of play friendships (*F*_(1,19)_ = 4.021, *p* = 0.059; Figure 4, bottom panel). Cohort was significant (*F*_(1,19)_ = 5.173, *p* = 0.035; Figure 3), with older females having higher AVP concentrations than younger females, but rank was not (*F*_(2,19)_ = 0.265, *p* = 0.770). We found neither sampling time point, parturition status, housing condition, nor blood sampling disturbance or draw times to significantly predict AVP levels (all* p* > 0.1). Beta coefficients for significant and trend-level effects are in Table 4.

**Table 4:** **Linear mixed model for females’ friendships and plasma vasopressin concentrations**.

Variable	*B*	Standard error
Birth cohort: 2002	161.17*	70.86
Birth cohort: 2003*^a^*	–	–
High Rank	−55.76	88.75
Middle Rank*^a^*	–	–
Low Rank	−41.11	66.48
Total Friendships	37.07*^b^*	18.13
Play Friendships	−63.44^ b^	31.64

^a^Reference category; ^b^0.05 < p < 0.10; *p < 0.05.

### Vasopressin model—males

**Figure 5:**
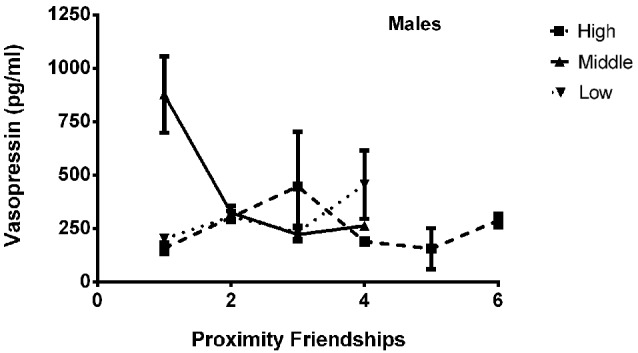
**There was a significant interaction between rank and the number of proximity friendships (*F*_(2,18.857)_ = 6.279, *p* = 0.008), such that middle-ranked males with only one proximity friendship had the highest AVP levels**.

The final model for vasopressin in males contained a number of significant friendship variables, including a significant positive association with the number of multiplex friendships (*F*_(1,22.166)_ = 7.140, *p* = 0.014) and significant negative relationships with the number of proximity (*F*_(1,22.319)_ = 7.361, *p* = 0.013) and play (*F*_(1,20.138)_ = 4.337, *p* = 0.050) friendships. We also found a positive association for subjects’ total number of friendships, which trended toward statistical significance (*F*_(1,20.802)_ = 3.370, *p* = 0.081). There was a significant interaction between rank and the number of proximity friendships (*F*_(2,18.857)_ = 6.279, *p* = 0.008; Figure 5), such that middle-ranked males with only one proximity friendship had the highest AVP levels. Cohort (*F*_(1,19.245)_ = 5.275, *p* = 0.033; Figure 3) and rank (*F*_(2,18.564)_ = 5.249, *p* = 0.016) were also significant, with older males having higher levels of AVP than younger males, and middle-ranked males having the highest AVP. In addition to these variables, measures associated with the blood draw procedures were significant predictors of AVP concentrations: disturbance time positively predicted AVP (*F*_(1,27.991)_ = 28.188, *p* < 0.0001), and draw time negatively predicted AVP (*F*_(1,37.113)_ = 4.722, *p* = 0.036). Neither sampling time point nor housing condition significantly predicted AVP levels (all *p* > 0.1). Beta coefficients for significant and trend-level associations are in Table 5.

**Table 5:** **Linear mixed model for males’ friendships and plasma vasopressin concentrations**.

Variable	*B*	Standard error
Birth cohort: 2002	117.61*	51.21
Birth cohort: 2003*^a^*	–	–
High Rank	−283.55*^b^*	147.46
Middle Rank*^a^*	–	–
Low Rank	−451.05**	139.74
Disturbance Time	0.30**	0.056
Draw Time	−0.72*	0.33
Total Friendships	55.29*^b^*	30.12
Multiplex Friendships	132.30*	49.51
Play Friendships	−91.21*	43.79
Proximity Friendships	−202.80**	46.71
High Rank × Proximity Friendships	123.52*	44.33
Middle Rank × Proximity Friendships*^a^*	–	–
Low Rank × Proximity Friendships	170.83**	49.25

^a^Reference category; ^b^0.05 < p < 0.10; *p ≤ 0.05 **p < 0.01.

## Discussion

Our study revealed that the number and quality of friendships experienced early in development by young rhesus monkeys predict the future functioning of their OT and AVP systems. These associations were complex, sex-specific, and often affected by rank, especially in females. In interpreting our findings, we note that our subjects’ friendship experiences were assessed at 1 year of age, which was 1–3 years prior to blood sampling. Thus, developmental processes, including negative or positive feedback on the OT and AVP systems, may have been reflected in peptide levels at the time that the samples were taken (Bales and Perkeybile, 2012).

Plasma OT was clearly more related to friendship in females than in males, as no friendship measure significantly predicted males’ OT concentrations. In females, OT concentrations were related to both reciprocal and play friendships. As mentioned in the Introduction section, reciprocity may be a key feature of relationships that are subserved by the OT system (Atzil et al., 2012; Schneiderman et al., 2012). In 3-year-old human children, salivary OT levels are positively correlated with reciprocal interactions with a best friend (Feldman et al., 2013). In adult human males, intranasal OT administration enhanced caudate nucleus response to reciprocated cooperation, which might indicate a more rewarding experience (Rilling et al., 2012). Reciprocal friendships are particularly significant in young rhesus monkeys, as they are among the highest quality friendships, and are most likely to persist over time (Weinstein and Capitanio, 2012); similar results have been found in humans (Gershman and Hayes, 1983; Bukowski et al., 1994). We found a quadratic, U-shaped relationship between plasma OT and the number of reciprocal friendships, which is in line with previous studies demonstrating U-shaped associations between OT and social behavior (e.g., Zhong et al., 2012), and suggests that opposing phenotypes of friendship involvement can generate a similar OT response. For females with very high numbers of reciprocal friendships, greater OT concentrations are in keeping with the abovementioned positive links between reciprocity and OT. The elevated OT levels that characterized females with no or only one reciprocal friendship, in contrast, is unclear; we speculate this finding may reflect a compensatory response of the OT system to a social stressor early in development (lack of high quality friends). This is consistent with the finding that plasma OT levels are higher in humans who experience gaps in their social relationships (Taylor et al., 2006), and may reflect an adaptive response which protects individuals from the deleterious physiological and behavioral effects of social isolation (Grippo et al., 2009). We also found an interaction between the number of reciprocal friendships and rank (Figure 1). The highest OT levels were displayed by middle-ranking females with no reciprocal friendships and high-ranking females with no or only one reciprocal friendship, suggesting that to these females, a lack of high quality friends may be a particularly powerful stressor. Low-ranking females, on the other hand, might presumably have lower expectations of receiving positive treatment from other animals, and not respond as much to the lack of reciprocal friendships. That dominance rank may influence an individual’s perceptions and expectations of her social environment and subsequent OT functioning is consistent with studies in humans showing OT’s relation to social behavior to be context-dependent, varying according to whether an individual perceives the social environment to be more or less safe (Olff et al., 2013).

We found a significant positive main effect of number of play friendships on OT concentrations in females, as well as a significant interaction with dominance rank. Social play typically requires an individual put him/herself in a vulnerable position, and Spinka et al. (2001) proposed that the vulnerability and loss of control inherent in play function to train individuals to physically and emotionally cope with unexpected stressful situations. The overall positive effect of involvement in play friendships on later OT concentrations supports this proposed function, as OT has been previously shown to decrease subjective ratings of stress (Heinrichs et al., 2003), possibly through its influence on parasympathetic control (Norman et al., 2011) and/or amygdala activity (Viviani et al., 2011). The relationship between OT, rank and play friendships in females (Figure 2) suggests, however, that only high-ranking females are especially sensitive to a lack of play friendships (as well as a lack of reciprocal friendships, see above), further supporting the idea that the greater OT concentrations in these high-ranked females may be a compensatory, adaptive response to a lack of social relationships which only emerges in a specific context (that of occupying a position of elevated social status).

In females, play friendships were also related to AVP levels, showing an inverse association that trended toward but did not reach significance (*p* = 0.059) (Figure 4, bottom panel; although graphically this appears to be another U-shaped relationship, the quadratic function was not significant). The same negative beta coefficient characterizing the relationship of AVP to number of play friendships in females was also evident in males, where it achieved significance. AVP is associated with many peripheral functions including water retention, increasing vascular constriction, physical activity (see below), and increasing HPA axis activity by potentiating the stimulatory effect of corticotropin-releasing hormone (CRH) on the secretion of adrenocorticotropic hormone (ACTH) from the anterior pituitary (Aguilera, 2011). Over the course of development, a greater number of play friendships might have led to lower levels of AVP through the stress-reducing effects of play. The fact that this association between play friendships and AVP achieved statistical significance only in males, however, suggests that play’s stress-relieving effects may be more potent in male than in female rhesus macaques, who spend less time playing overall (Weinstein and Capitanio, 2008). Only a small handful of studies—primarily limited to rodents—have specifically investigated AVP’s relation to play (Cheng et al., 2008; Cheng and Delville, 2009; Veenema and Neumann, 2009; Wang et al., 2012), and the mechanisms by which play may regulate and be regulated by the AVP system are therefore not fully understood, and warrant further investigation.

In males, higher plasma AVP was significantly associated with a greater number of multiplex friendships, which are characterized by a preference displayed toward a peer for more than one type of social interaction. AVP has been associated in many social contexts with a willingness to engage proactively (Carter et al., 1995; Bosch and Neumann, 2012), and in some contexts and dosages it is anxiolytic (Dharmadhikari et al., 1997). It is therefore not surprising to see it associated with higher numbers of multiplex friendships, in that more proactive animals might be likelier to initiate consistent social interactions with a greater number of peers in a variety of behavioral domains. High plasma AVP also tended to be positively predicted by the total number of friends in both females (Figure 4, top panel) and males, though the associations were not statistically significant (*p* = 0.055 for females and *p* = 0.081 for males). Although these trends support the aforementioned notion that high AVP may relate to a more socially proactive phenotype, their failure to reach significance suggests that the total number of an individual’s friends may not be the most relevant factor to neuropeptide functioning, and that—at least for males—it is involvement in behaviorally complex (i.e., high quality) friendships that plays a key role in the development of the AVP system.

In males, but not females, AVP levels were significantly predicted by involvement in proximity friendships, suggesting differential importance of proximity friendships by sex. Proximity friendships are characterized by spending significant amounts of time together in a relatively sedentary state. Males with more proximity friendships had decreased AVP levels, consistent with this neuropeptide’s expected inverse relationship with physical inactivity. The relationship between rank, proximity friendships, and AVP was complicated, with middle-ranking animals who had only one proximity friendship—the lowest number of proximity friendships held by any animal—showing especially high levels of AVP (Figure 5). Middle-ranked monkeys hold a unique status in their social groups, as they are dominant and subordinate to approximately the same number of their group-mates, and consequently must be equally prepared to exhibit either one of two opposing responses to a conspecific (dominance or submission). Middle-ranked individuals are more attentive to a variety of social situations as compared to their high- and low-ranking counterparts (Haude et al., 1976; Capitanio et al., 1985), and heightened social attention may be necessary for such behavioral flexibility. As social attention and perception may relate to AVP functioning (Thompson et al., 2004, 2006), we speculate that in our male subjects, the high attentional demands of being middle-ranked are reflected by high AVP concentrations (recall that middle-ranked males had higher AVP overall), which may decrease as a result of the buffering effects of having more proximity friends. The manifold associations between different friendship types and plasma AVP in males suggests that in rhesus macaques, as in humans, AVP’s role in social relationships reflects diverse social processes (Gouin et al., 2012), which may also be influenced by an individual’s social environment (i.e., dominance rank).

In males, disturbance time positively related to plasma levels of AVP, while draw time was negatively related. The positive association between disturbance time (the time that elapsed between the opening of the door to animals’ enclosure and the time the needle was withdrawn) and AVP suggests that AVP was secreted in response to physical activity and/or the stress of the capture procedure in males. At the CNPRC, net capturing a single monkey causes all of the monkeys in a particular corral to run several laps around its periphery. Previous studies in both humans and animals have found that plasma AVP levels show an immediate surge in response to exercise (Alexander et al., 1991), and the degree to which AVP levels rise is determined by exercise intensity (Convertino et al., 1981; el-Sayed et al., 1990) and duration (Coiro et al., 2010; Reza et al., 2011). AVP secretion following the experience of running and net capture may in turn stimulate the release of ACTH, thus activating the HPA axis (Aguilera, 2011). Conversely, draw time reflects the amount of time our subjects spent being restrained for the blood draw, and therefore physically inactive, and the inverse relationship between draw time and AVP suggests that AVP levels began to drop as soon as the animal was captured. Similarly, in humans, AVP levels begin to drop almost immediately upon exercise cessation (Ferrari et al., 1991; Wittert et al., 1991). To our knowledge, ours is the first study to demonstrate a relationship between physical activity and AVP in nonhuman primates, though we found this effect only in males. This sex-specific effect may reflect the life-history difference that while females remain in their natal group their entire lives, males emigrate from their troop upon reaching puberty, and wander until they find a new troop to join (Lindburg, 1971). AVP may thus be an important mechanism involved in the regulation of male emigration; consistent with this interpretation is the fact that our male subjects were approaching or had already reached adolescence, and nearly ready to commence emigrating had they been living in the wild. Our results therefore indicate that measures of physical activity may be important to include in any future studies of AVP and social relationships.

We also found a relationship between birth cohort and both OT and AVP, with AVP higher in both older males and older females, and OT higher in older females (Figure 3). Note, however, that we did not find significant differences in either OT or AVP between the first and second blood sampling time points, suggesting that neither OT nor AVP concentrations show seasonal variation in this species, and that any developmental changes in our subjects that occurred between these two time points were not substantial enough to significantly alter neuropeptide concentrations. Our first blood sample was taken from 2.5 and 3.5 year olds, thus the 2.5 year olds were pre-pubescent while the 3.5 year olds were peri/post-pubescent (and some females were mature enough to be in the very early stages of pregnancy). These two cohorts thus represent a contrast between juvenility-early adolescence and adolescence-early adulthood. There is only limited evidence for changes in plasma OT levels with aging (Ebner et al., 2013), however, since OT levels are estrogen-dependent (Amico et al., 1981), a rise might be expected with reproductive maturation in females. Similarly, higher AVP levels in the older cohort may be associated with maturation of the reproductive axis; AVP is androgen-dependent (De Vries and Villalba, 1997), and testosterone would likely be higher in peri/post-pubescent than in pre-pubescent males (Bernstein et al., 1991). We note that at both blood sampling time points, pregnancy/having an infant had no effect on either neuropeptide.

Friendships are especially important to study in some special populations such as children with autism (Rowley et al., 2012). Children with autism display fewer reciprocal friendships (Kasari et al., 2011), and these lower quality friendships in some cases caused even higher anxiety than a lack of friends (Mazurek and Kanne, 2010). Play dates can be beneficial to social functioning in children with autism (Frankel et al., 2011), especially with parent-assisted supervision (Frankel and Whitham, 2011). Given both the large number of associations between OT, AVP, and autism (Carter, 2007; Jacob et al., 2007; Gregory et al., 2009, 2013; Feldman, 2012), and the current initiatives to use intranasal OT as a therapeutic for autism (Anagnoustou et al., 2012; Bakermans-Kranenburg and Van Ijzendoorn, 2013), it will be important for future studies to consider friendships and how these treatments affect the quality of this very salient real-life measure of functioning in children with autism.

Overall, we found OT and AVP to relate to friendship in very sex-specific ways. Sex differences in friendship involvement have been well documented in children (Kon and Losenkov, 1978; Benenson et al., 1998; Hardy et al., 2002; Benenson and Christakos, 2003; Benenson and Alavi, 2004; de Guzman et al., 2004), and have been similarly demonstrated in rhesus macaques (Weinstein and Capitanio, 2008, 2012). The examination of the sex-specific ways in which OT and AVP relate to social relationships in nonhuman primates has thus far been rarely studied, but may be particularly important because of potential translational relevance. While autism is primarily a condition found in males (CDC, 2012), 1 in 252 American girls are also affected. So far, clinical trials with OT have been carried out mainly in males (Tachibana et al., 2013; Dadds et al., 2014), or in samples with insufficient numbers of females to detect sex differences in treatment efficacy (Anagnoustou et al., 2012). Plasma OT and AVP show sex-specific relationships to autism symptoms (Miller et al., 2013). Social context is also potentially important to the actions of OT (Bartz et al., 2011), suggesting that sex differences in friendships could interact with sex differences in reactions to therapeutic OT.

It is important to note that although we found several significant associations between early friendship involvement and later neuropeptide concentrations, it is impossible to determine the precise causal relationships between these variables. Given that our friendship measures preceded blood sampling by 1 or more years, we believe it likely that early friendship involvement produced lasting effects on neuropeptide functioning. However, as mentioned in the Introduction section, it is certainly possible for early neuropeptide functioning to influence an individual’s propensity to form friendships as well (e.g., Bales et al., 2007b). Previous research suggests that peripheral neuropeptide concentrations reflect both trait-level characteristics (i.e., personality or temperament) and state-level factors (i.e., aspects of an individual’s social environment and interactions; Strathearn et al., 2012), thus the peripheral concentrations in our subjects could have similarly been a product not only of the state-level variables of friendship involvement, but of trait-level variables such as temperament, which our earlier studies have shown to influence rhesus macaque friendships (Weinstein and Capitanio, 2008, 2012). Examining the relationship between temperament and neuropeptide concentrations in this species is a direction that we are currently pursuing.

Finally, there is a gap in the literature on neuropeptides and social relationships with regard to friendships. This represents a future direction that should be explored, for several reasons. First of all, friendships are distinct from attachment relationships (Mason and Mendoza, 1998), but how the physiological substrates differ is not known. Friendships are important for children’s healthy development, both neurotypical and those with developmental disorders. Friendships may be more pervasive than attachment relationships, and are very important in predicting long-term health outcomes, which particularly calls for further investigation of the role of OT in social buffering. Lastly, in a recent review of sociality and neuropeptides, Goodson (2013) points out that sociality is a multifaceted phenomenon which is often mistakenly considered homogeneous and therefore not parsed into its various components when examining the underlying roles of OT and AVP. Specifically teasing apart the roles played by OT and AVP not only in the general phenomenon of friendship, but in specific types of friendships that vary in quality, is therefore critical in order to understand how neuropeptides relate to each of the wide-ranging aspects of sociality that evolved in both human and animal species.

## Author contributions

Tamara A. R. Weinstein, John P. Capitanio, and Karen L. Bales designed the study and analyzed the data. Tamara A. R. Weinstein conducted the research with critical assistance from Nicole Maninger and Caroline M. Hostetler. The manuscript was written by Tamara A. R. Weinstein and Karen L. Bales, and edited by all authors. John P. Capitanio and Karen L. Bales supervised the project.

## Conflict of interest statement

The authors declare that the research was conducted in the absence of any commercial or financial relationships that could be construed as a potential conflict of interest.
